# The Fatty Acid Content, Health Lipid Indices, and Instrumental, Histological, and Sensory Quality of Hare Meat (*Lepus europaeus* Pallas)

**DOI:** 10.3390/foods14020310

**Published:** 2025-01-17

**Authors:** Gabriela Frunză, Marius-Mihai Ciobanu, Otilia Cristina Murariu, Răzvan-Mihail Radu-Rusu, Paul-Corneliu Boișteanu

**Affiliations:** 1 Department of Food Technologies, Faculty of Agriculture, “Ion Ionescu de la Brad” Iași University of Life Sciences, 8 Mihail Sadoveanu Alley, 700489 Iasi, Romania; gabriela.frunza@iuls.ro (G.F.); marius.ciobanu@iuls.ro (M.-M.C.); 2 Department of Animal Resources and Technology, Faculty of Food and Animal Sciences, “Ion Ionescu de la Brad” Iași University of Life Sciences, 8 Mihail Sadoveanu Alley, 700489 Iasi, Romania; 3 Department of Control, Expertise, and Services, Faculty of Food and Animal Sciences, “Ion Ionescu de la Brad” Iași University of Life Sciences, 8 Mihail Sadoveanu Alley, 700489 Iasi, Romania; paul.boisteanu@iuls.ro

**Keywords:** meat, hare nutritional value, health lipid, quality

## Abstract

The aim of this work was to characterize the quality of meat from hares (*Lepus europaeus* Pallas), namely, the fatty acid content, health lipid indices, and instrumental, histological, and sensory profiles by gender and muscle type (*Longissimus dorsi/LD* vs. *Semimembranosus/SM*). The ΣPUFA/Σ SFA was higher for males, with an average value of 1.62/1. The Σn6/n3 ratio was elevated for males, with a mean value of 5.34/1. The mean meat essential fatty acids were 41.94%, the desirable fatty acids were 77%, and the polyunsaturation index was 6.09. Moreover, the atherogenic index was 0.72, the thrombogenic index was 0.71, the hypocholesterolemic/hypercholesterolemic (h/H) fatty acids ratio was 3.30, and the nutritive value index was 1.35. After the sensory analysis, the LD muscles showed higher scores in males for overall appreciation (4.20 vs. 3.95) but higher scores in females for SM muscles (4.14 vs. 4.00). Shear force was influenced by the ratio between muscle and connective tissue, and the proportion of collagen and protein was related to the number of muscle fibers. Muscle and connective tissues are inversely proportional, and their ratio is an indicator of the textural and mechanical properties of the analyzed samples. Hare meat is an appreciated resource for consumers in terms of its sensory, instrumental, and nutritional values, and it has a higher value than that obtained from livestock species or other wild animals (more valuable proteins, lower fat content, and better health lipid indices).

## 1. Introduction

Recently, people have become conscious of the relationship between food intake and the state of well-being. Rational nutrition must provide the human body with the energy necessary for life, as well as the vitamins, minerals, and especially proteins that constitute the foundation of living beings. Thus, people are motivated to find healthier food alternatives by ingesting foods low in saturated fat but with a high protein content [1]. Rabbit and hare meat is a healthy alternative. The meat from game species has an increased attractiveness in terms of product intake due to its beneficial properties, which address humans’ desires for foods that have a complete and complex nutritional value [2,3]. Also, the meat of game species contains complex compounds that lead to the creation of distinct aromatic characteristics [3], highly appreciated by connoisseurs. The essential quality of hare meat is represented by its complete nutritional value [4]; the intake of this meat can improve people’s health of because of the high content of highly valuable proteins, unsaturated lipids (high content of n6 and n3 fatty acids) [3,4], vitamins, and minerals.

The brown hare (*Lepus europaeus* Pallas) does not live in just one country and is found in a multitude of places that provide suitable living conditions. It has a different nutrition profile specific to the geographical area where it lives [5], which can influence the proprieties of its meat.

The hare is among the best-known species of small size for hunting [6,7,8,9,10,11,12,13,14,15,16,17,18,19,20,21,22,23]. In Italy, the hare can be found in breeding farms, where it is grown in order to repopulate hunting and protected areas [6,7]. Hare meat has all the essential conditions to be included in human alimentation, thanks to its sensory properties [7], richness in valuable proteins containing essential amino acids, low fat content with a high concentration of unsaturated fatty acids [8], and content of bioavailable minerals and vitamins [9], although its energetic value is low [6,10], similar to that of lean meat. From the point of view of its high iron and myoglobin content, hare meat is considered a red meat [6,13]; it is, therefore, an alternative to other red meats, the production of which results in higher CO_2_ emissions. However, an inconvenience to its use would be the limited access to this valuable meat, which depends on the seasonality of hunting. Usually, hares consume a multitude of plants, herbs, and cereal grains that have high variability in terms of availability, quality, and chemical composition (depending on the atmospheric conditions and season); this has a considerable impact on meat quality, which may fluctuate as a result [24]. The analysis of the quality of hare meat at the level of the specialized literature is relatively limited [6,7,8,9,10,11,12,13,14,15,16,17]. Only five publications describe the quality of hare meat obtained by shooting in Austria [10,11], Croatia [12], Slovakia [13], and Lithuania [17]; three different publications present the analysis of the quality of hare meat obtained in breeding farms in Italy [6,15] and Poland [16]. The population of *Lepus europaeus* in many European countries has declined because of multiple factors [12,17,19,20,21,22,23,24]; therefore, there is a need to regulate their populations [12,17,23]. Currently, in Romania, but also in other countries such as Italy and Lithuania, the brown hare is not excessively hunted at volume, and hunting is prohibited in some areas. The annual abundance of hares fluctuates by 5–10% [17,24]. For research purposes, it has been observed that the hare can be exploited in captivity [6,16,25,26]; in Italy and Poland, it is being bred along with other wild animal species in order to restore the totality of indigenous specimens [27] with the objective of repopulation, providing additional game [28,29] and sources of nutritional meat [29,30]. According to the FAO, the estimated population worldwide of hare and wild rabbits was 158,417 in 2022, and 2,602,820 in the past ten years [31]. In Romania, the estimated population of hares (the harvest quotas) was 96,904 in the 2023–2024 hunting season and grew to 98,421 in the 2024–2025 season [32]. The lack of data (at the European and especially at the national level) on the quality analysis of hare meat is what led us to perform this investigation. This paper is part of a larger study that characterizes domestic rabbit meat (from the Giant Belgian breed) and that of the brown hare (*Lepus europaeus* Pallas). The purpose of this paper was to perform meat characterization of hares (the fatty acid content and instrumental, histological, and sensory quality analysis) from Iasi County, Romania, by gender and muscular groups.

## 2. Materials and Methods

### 2.1. The Animals

**Figure 1:**
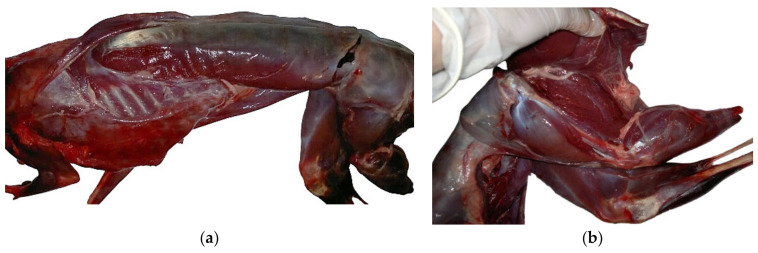
Meat sampling: (**a**) *Longissimus dorsi* (LD) and (**b**) *Semimembranosus* (SM) muscles.

Three hunting funds (from Iasi County, Romania) have provided the animals for this study, i.e., 33 hares (15 males and 18 females), who were shot on different days during the November to January. The hares were aged about 9.5 months, as indicated by the Stroh method [33] (the length of epiphyseal ossification in ulna), and had an average carcass weight of 4.2 kg. Right after shooting, the hares were skinned and the gastro intestinal tract and abdominal adipose tissue were removed; then, the eviscerated carcasses were sent to the laboratory and stored at 4 °C prior to analysis. Meat sampling (Figure 1) was carried on two different muscular groups, *Longissimus dorsi* (LD) and *Semimembranosus* (SM), based on their representativeness for size and metabolic, chemical, histological, and instrumental features.

Half of each individual body was used for chemical analysis and the other half for histological and instrumental/textural assessments. Lipids and fatty acids were analyzed on muscle samples that were chopped and homogenized. 

### 2.2. Chemical Properties of Hare Meat

An electric chopping machine was used in homogenously preparing the samples for chemical analyses [1]. Lipids, proteins, and collagen contents were analyzed using a Food-Check Spectrophotometer (Near InfraRed, Bruins Instruments, KPM Analytics, Bad Camberg, Germany) [34].

### 2.3. The Fatty Acids Analysis

Fatty acid content, as described by Frunza et al. 2023 [1], was analyzed with a FOSS 6500 Near InfraRed spectrophotometer (FOSS Analytics, Hilleroed, Denmark). The samples were lyophilized at −110 °C for a period of 24 h [1] using a freeze dryer (CoolSafe ScanVac, LaboGene co., Odense, Denmark), weighed with a technical scale again, and stored at −80 °C [35]. The following contents were analyzed: the main saturated fatty acids (SFAs), C14:0–C18:0 [1]; the monounsaturated fatty acids (MUFAs), n7 and n9 (16:1n-7, C18:1n-7, and C18:1n-9 [1]); and the polyunsaturated fatty acids (PUFA), n3 and n6 (C18:2n-6 to C22:6n-3 [1]).

### 2.4. Calculation of Health Lipid Indices

The health lipid indices were evaluated by summing the saturated fatty acids (SFAs), monounsaturated fatty acids (MUFAs), and polyunsaturated fatty acids (PUFAs) to identify the essential fatty acids (EFAs), as indicated by Chen et al. [36]. The polyunsaturation index (PI) was calculated following the method outlined by Timmons [37]. Additionally, the atherogenic index (AI) and thrombogenic index (TI) were derived using the Ulbricht and Southgate method [38]. The hypocholesterolemic-to-hypercholesterolemic ratio (h/H) was obtained following Fernandez et al.’s [39,40,41,42,43,44] method. The nutritive value index (NVI) and desirable fatty acids (DFAs) were calculated according to Wereńska et al. [45]. Relations (1)–(7) were applied in the calculation of the indices listed above:

PI = C18:2n-6 + (C18:3n-3 × 2)(1)

AI = [(4 × C14:0) + C16:0 + C18:0]/MUFA + PUFA n-6 + PUFA n-3;(2)

TI = (14:0 + 16:0 + 18:0) / [(0.5 × MUFA) + (0.5 × n-6 PUFA) + (3 × n-3 PUFA) + (n-3 PUFA/n-6 PUFA)];(3)

h/H = (C18:1 + PUFA)/(C14:0 + C16:0);(4)

NVI = (C18:0 + C18:1)/C16:0;(5)

DFA = ∑MUFA + ∑PUFA + C18:0.(6)

EFA = C18:2n-6 + C18:3n-3 + C20:4n-6(7)

### 2.5. The Instrumental, Histological, and Sensory Qualities of Hare Meat

For textural assessments, the samples were sealed under vacuum and treated at 80 °C in a water bath, with a slow movement of the content, for one hour. They samples were then cooled to 20 ± 2 °C and subsequently preserved at 4 °C prior to analysis. Four to six prisms of 1 cm height × 1 cm width × 2 cm length were cut for each muscle, with the fibers (myocytes) parallel with the longitudinal axis [36,37]. A Warner–Bratzler cell texturometer (TA-XT2I, Stable Micro System, Surrey, UK) was used to at a perpendicular cutting speed of 5 mm/second. The total shear force (kg/cm^2^), firmness (kg/s × cm^2^), and area (kg × s/cm^2^) were measured and compared by gender and muscle type.

Muscles color analysis was carried out with a CR-300 Chroma Meter (Konika Minolta, Ramsey, NJ, USA), using the CIELAB system (L = brightness coordinate; A/a* = red index; and B/b* = yellow index). To avoid the penetration of light through muscles and imminent errors that may appear, samples of 1.5–2.5 cm in height were first exposed to air for 30–40 min to become oxygenated. Prior to analysis, the colorimeter was calibrated to the standardized values (L = 98.19; a* = 0.0; b* = 1.93). The device surface was cleaned with ethyl alcohol after each reading to avoid possible errors that may appear.

Histological measurement were carried on the samples processed through paraffin inclusion and slicing technique, using a Rotary automatic microtome THERMOSCIENTIFIC HM355S (Fisher Scientific, Leicestershire, United Kingdom) microtome, anatomical and histological working tools, coloring baths, a Motic MWB1-223 (Motic Europe S.L.U., Barcelona, Spain) microscope, and Image Motic Plus 2.0ML software (Motic Europe S.L.U., Barcelona, Spain). Also, the following reagents were used: formalin (10%), ethanol (85°, 90°, and 96°), amyl alcohol, toluene, benzene, xylene, Harris hematoxylin dyes, color fixers, and Mayer albumin. Optical data were acquired and processed using the microscope software and ordered into a database by the Microsoft Excel 2016 (Microsoft Co., Redmont, WA, USA).

The sensory quality of hare meat was analyzed by 23 trained tasters, with pre-tasting sessions (according to the ethics and informed consent approval 1112/05.XII.2023). The panelists rinsed their mouths between the randomized samplings of meat, which was served at an ambient temperature of 21–22 °C and under white light (according to the standardized method). Adapted after Ariño et al., 2007 [46], the panelists filled in assessment sheets of the sensory characteristics of hare meat (Table 1) using a five-point hedonic scale (1 point = unfavorable characteristics; 5 points = fully meets the requirements).

### 2.6. Data Analysis

The obtained results were statistically processed (arithmetic mean; SEM—standard error of the mean; V%—coefficient of variation) using Excel software. To assess the significance of gender and muscle type differences, the results of the analysis of males and females (33 results) were compared using the Student (t) test in GraphPad Prism 9.4.1. software (GraphPad co. ltd., Palo Alto, CA, USA).

## 3. Results

### 3.1. Chemical Properties

The lipid, protein, and collagen contents of the samples are presented in Table 2.

The average values of lipids, proteins, and collagen were close between genders, with small though not statistically significant (p > 0.05) differences for lipids: 1.52 for males vs. 1.78 for females in LD muscles and 1.81 for males vs. 2.03 in SM muscle for females.

### 3.2. The Fatty Acids Content

In *Longissimus dorsi* samples, the most frequently occurring fatty acid was linoleic acid/C18:2n-6 (592.93 mg/100 g meat), followed by oleic acid/C18:1n-9 (353.12 mg/100 g meat), both in males (Table 3).

In *Semimembranosus* samples, the same fatty acids were found in the highest proportions (C18:2n-6, 557.02 mg/100 g meat; C18:1n-9, 308.08 mg/100 g meat).

The highest SFA content was identified in the LD muscles of males, with palmitic acid/C16:0 being the highest (322.87 mg/100 g).

Higher mean values of fatty acids were found in males (with small differences compared to females) in the LD muscles (Table 3).

Predominantly significant differences between genders (*p* < 0.001) were highlighted in SM samples, while in LD samples, the differences were mostly significant (*p* < 0.05) or even insignificant (*p* > 0.05).

### 3.3. Health Lipid Indices of Hare Meat

Table 4 presents the total amount of saturated, monounsaturated, and polyunsaturated fatty acids and health lipid parameters of hare meat.

In males, a higher SFA content was found for both muscle groups (474.25 mg/100 g in LD and 449.51 mg/100 g in SM) compared to females (465.19 mg/100 g in LD and 394.54 mg/100 g in SM).

Total MUFAs (mg/100 g) was higher in males on average (359.37 vs. 284.70 in females). Total PUFAs was also higher in males (783.97 vs. 661.35 mg/100 g), probably based on the differences related to their diets. The ΣPUFA/Σ SFA was better in males (1.71/1 vs. 1.54/1 in LD and 1.69/1 vs. 1.53/1 in SM) and showed the same trend for the average value of the meat (1.70/1 in males vs. 1.54/1 in females (average value of 1.62/1 for hare meat)).

The ΣPUFA n6/n3 ratio was also higher in males (5.62/1 vs. 5.02/1 in LD and 5.72 vs. 5.01 in SM), with a ratio of 5.67/1 in males and 5.01/1 in females (average value of 5.34/1 for hare meat).

PI was better in males’ LD muscles (6.99 vs. 5.92 in females) and presented a wider amplitude in SM samples (6.58 in males vs. 4.95 in females).

The DFA content (mg/100 g) was higher in males (1307.28 vs. 1121.33 in females for LD and 1204.57 vs. 962.91 in females for SM), with averages of 1255.93 (males) vs. 1042.12 (females). The calculated %DFA was better in males vs. females (78.59% vs. 75.44% in LD and 77.86% vs. 76.10% in SM), with a mean of 77.00 in hare meat.

Calculated AI was higher in males (0.81 vs. 0.72 in LD and 0.76 vs. 0.61 in SM) while the TI presented the same trend, with higher values in males (0.77 vs. 0.72 in LD samples and 0.72 vs. 0.61 in SM).

The h/H indices were lower in females (3.00/1 in vs. 3.64/1 in males’ LD and 3.09 in females vs. 3.47/1 in males’ SM). The average values (for both muscles) of the h/H ratio was also lower in females (3.04 vs. 3.56 in males), indicating a greater hypocholesterolemic influence if consumers only use the meat of male hares in alimentation.

In females, the NVI was lower compared to that in males (1.19 vs. 1.54 in LD and 1.27 vs. 1.41 in SM), with an average between 1.23 (females) and 1.47 (males) and a general value of 1.35 in hare meat.

Total EFAs (mg/100 g) was higher in males vs. females (709.37 vs. 614.18 in LD and 668.53 vs. 513.37 in SM), and therefore, the proportion of EFAs (%EFA) was higher in males (42.65% in LD and 43.21% in SM) than in females (41.32% in LD and 40.57% in SM), with a mean of 41.94%/hare meat.

### 3.4. The Instrumental Assessment of Hare Meat

#### 3.4.1. The Textural Traits

The results in Table 5 refer to the measured shear force/cutting force, firmness, and area under the curve.

The highest mean value for total shear force was observed in females’ SM (4.32 kg/cm^2^) while in males, this value reached 3.29 kg/cm^2^ (*p* < 0.001). In LD muscles, males presented a higher shear force (2.72 kg/cm^2^) than females (2.01 kg/cm^2^).

In the firmness analysis, the greatest mean value was found in females’ SM (1.54 kg/s × cm^2^ vs. 1.16 kg/s × cm^2^ in males; *p* < 0.001/***). The LD samples showed greater toughness in males (1.17 kg/s × cm^2^) vs. females (0.92 kg/s × cm^2^).

The mechanical work (area) necessary to cut the female SM muscle samples was higher (8.82 kg × s/cm^2^) while male SM muscles had a lower value of 5.45 kg × s/cm^2^. The same trend was observed in LD muscles, with increased values in males (4.69 kg × s/cm^2^) compared to females (3.95 kg × s/cm^2^).

#### 3.4.2. The Color Traits

Table 6 presents the color parameters of hare meat, obtained using the CIELAB system.

In LD samples, close values were found for L*/lightness in females (28.55) and males (28.94). The red parameter (a*) had different values: 13.58 in females, and 12.71 in males (*p* < 0.001). The yellow parameter (b*) was higher for males (13.28) compared to females (12.77) (*p* < 0.001).

In SM samples, the gender differences consistently affected the muscle color, such as in terms of brightness index (L* = 32.11 in females vs. L* = 31.38 in males), red index (a* =6.84 in females vs. 6.11 in males), and yellow index (b* = 10.02 in females vs. 9.75 in females).

#### 3.4.3. The Histological Traits of Hare Meat

The histological dimensional parameters of hare meat (large diameter (μ), small diameter (μ), and mean diameter (μ) of myocytes; the ratio between diameters, BD/sD (μ); the muscular fibers area (µ^2^); and myocytes cross-sectional area (µ^2^)) are presented in Table 7.

#### 3.4.4. The Main Categories of the Muscle Tissues in Hares

The main categories of muscle tissues in hares are presented in Table 8.

The muscle tissue proportion was higher in males for both muscles (71.01% vs. 69.40 in LD and 64.92 vs. 63.82 for SM muscles). Therefore, the number of muscle fibers, the density, and the ratio of MT/CT were higher, and in consequence, only the connective tissue was lower in males. Significant differences in muscle fiber density between females and males occurred only for LD.

#### 3.4.5. The Sensory Indicators of Hare Meat

**Figure 2:**
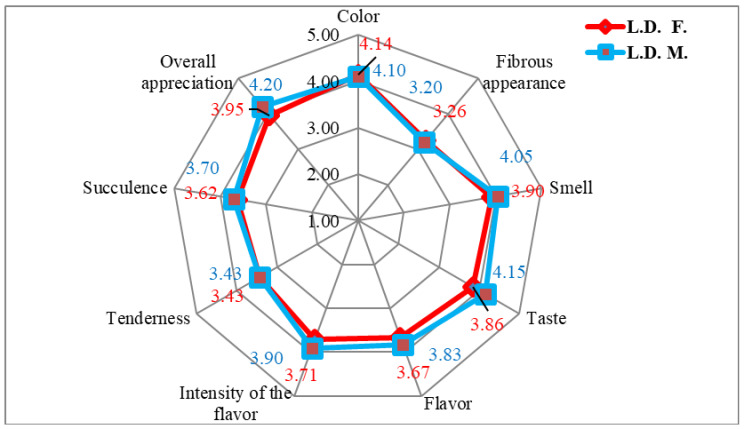
The sensory appreciation of LD muscles from hares.

The mean scoring of LD samples was higher in females compared to males (Figure 2) for color (4.15 vs. 4.10) and fibrous appearance (3.26 vs. 3.20). Higher grades were obtained in males for smell (4.05 vs. 3.90), taste (4.15 vs. 3.86), flavor (3.88 vs. 3.67), the intensity of flavor (3.90 vs 3.71), succulence (3.70 vs 3.62), and overall appreciation (4.20 vs 3.95). The tenderness had the same value for males and females (3.43).

**Figure 3:**
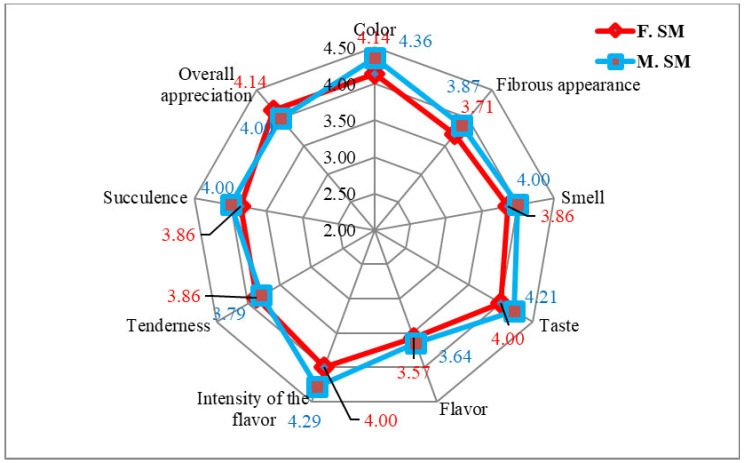
The sensory appreciation of SM muscles from hares.

In SM muscles (Figure 3), the males obtained higher grades in color (4.36 vs. 4.14), fibrous appearance (3.87 vs. 3.71), smell (4.00 vs. 3.86), taste (4.21 vs. 4.00), flavor (3.64 vs. 3.57), the intensity of flavor (4.29 vs. 4.00), and succulence (4.00 vs. 3.86). Higher scores were obtained by females for the tenderness (3.86 vs. 3.79) and overall appreciation (4.14 vs 4.00).

## 4. Discussion

This study presents various results regarding the quality of hare meat, one of the most abundant game species in Europe. Unfortunately, we did not find similar data in the literature for all the parameters analyzed in the current study.

Global food scarcity has motivated humanity to find new quality sources of nutrients (especially lipids and proteins) [1,47]. Furthermore, the frequent and excessive overconsumption of unhealthy foods leads to metabolic diseases, obesity, hypercholesterolemia, type 2 diabetes, hyperuricemia, hormonal disorders such as insulin resistance, leptin and ghrelin imbalances, and cardiovascular diseases, among others.

Meat from game species has been consumed since the most ancient times, and the consumption of unconventional animal species is increasing now globally [48]. In Romania, hunting has been perceived for over 50 years as a rational activity, carried out in order to manage, using hunting weapons, the balance of nature, in particular to maintain the balance between predatory and herbivorous game species and between game species in general and their environment, preventing the depletion of resources and invasive species phenomena.

Hare meat can be a solution for food insecurity (guaranteeing access to sufficient proteins and lipids with high biological value). Hare and rabbit meat [1,47,48,49,50,51,52,53] can align with the desires of humans as a functional food rich in essential amino acids, essential fatty acids, and bioavailable vitamins and minerals. In addition, game meat offers a special culinary experience, bringing a wild and authentic touch to culinary preparations, especially for cooking enthusiasts who appreciate refined dishes.

Game hunting has remained an important activity in many countries, not only as a way to provide food, but also as a sport which enjoys special consideration and is deeply rooted in European culture, tradition, and heritage [54]. These hunting practices are adjusted according to wildlife populations, thus ensuring environmental sustainability. Europe has above seven million registered hunters [55], most of them from France (1.3 million), Spain, the United Kingdom, and Italy. For proper management of species populations, hunting quotas are used [48]. The breeding of hares (*Lepus europaeus* Pallas) in captivity has proven to be a favorable way of repopulating geographical areas with a deficit of these animals (such as in Italy) and of capitalizing on the production of meat with better quality compared to other species [7,15]. This good example should adopted by other countries.

### 4.1. Chemical Composition and Health Lipid Indices of Hare Meat

#### 4.1.1. Chemical Properties

The chemical properties of hare meat are presented in Table 2.

Our study reported lower or similar protein quantities in hare meat compared to other studies from Croatia, Slovakia, Italy, Poland, and Lithuania [12,13,15,16,17]. In hares hunted in Croatia, the chemical composition highlighted smaller quantities of lipids (1.09%) and higher levels of proteins (23.08%) [12]. Values pretty similar to ours were found in hunted hare meat from Lithuania [17], with 21.90% proteins and 1.87% lipids in *Longissimus thoracis et lumborum* and 21.58% proteins and 2.07% lipids in *Biceps femoris* (BF muscles), and from Italy for sub-adult farmed hares [15], with an average of 21.55% proteins and 1.56% lipids. A study from Poland [16] reported an average of 22.56% crude proteins and 3.21% lipids in farmed brown hares (the higher amount of lipids than in this paper is due to the limitation in movement of the animals that were raised in cages and possibly to a diet richer in energy).

#### 4.1.2. The Fatty Acids Content and Health Lipid Indices of Hare Meat

Table 3 presents the fatty acids content in meat, and Table 4 shows the health lipid indices of hare meat (for LD and SM muscles), knowing that the quality of nutrients provided by food is a key element in evaluating the health status changes and development of human consumers [56].

According to some authors, a 0.45 or higher ratio of PUFAs/SFAs in food is recommended to limit cardiovascular illness [51]. The sanogenity (health-generating potential) of dietary lipids can be also evaluated by the occurrence of cholesterol neo-genesis in consumers’ hepatocytes and is evaluated as a ratio of hypocholesterolemic to hypercholesterolemic (h/H) fatty acids [57].

The thrombogenicity index (TI) shows the linkages between prothrombogenic (saturated) and anti-thrombogenic fatty acids [38]. The TI gives clues about the possible formation of clots in patients’ circulatory systems. To ensure cardiovascular protection and prophylaxis of atherosclerosis and thrombosis, the values of TI and AI in foods must be lower than 1.0 [58]. For a healthy lifestyle and diet, rabbit meat is recommended [59,60,61,62,63,64,65,66,67]. In some studies, rabbit meat had superior AI (0.52–0.72), and thus the TI (0.59–1.14) was higher compared with the values evaluated as safe for human consumption [60]. In this study, the AI and TI had smaller mean amounts, showing healthier indices for consumers (a mean of 0.72/ meat). The AI (0.72 vs. 0.81 in LD and 0.61 vs. 0.76 in SM samples) and TI (0.72 vs. 0.77 in LD and 0.61 vs. 0.72 in SM samples) of female hares were lower than males, suggesting that the meat of female hares is healthier. After Dal Bosco et al. 2024 [47], the best TI value was found in meat with horse fat (0.76 and 0.91 in low-fat meat). In our study, the TI value was smaller, at 0.71 mean/ meat, than in horses. Moreover, pork steak shows the worst values, characterized by high levels of SFAs [47].

The intake of red meat is commonly said to have a negative impact on health status [68,69]. Relevant literature shows a weak link between the consumption of unprocessed red meat and health [70], and the data do not allow per se the confirmation of an association between unprocessed red meat and cardiovascular illness [71,72].

The biological activity of hare and rabbit meat is based on fatty acids, which are implicated in consumers’ cardiovascular health [1]. A source of healthy dietary fats is rabbit meat, offering an excellent nutritive value [49,53], recommended for patients with hypertension (as it has a low sodium content), hyperlipidemia (having dietetic properties) [49], and cardiovascular and cerebrovascular diseases [71,72,73,74,75,76]. An index put into practice to evaluate the effect of food on cardiac and vascular health is the PUFA/SFA ratio [71]. It is hypothesized that PUFAs in the diet can lower low-density lipoprotein (LDL) cholesterol and thus decrease serum values; in relation to these benefits, the higher this ratio, the higher the desired contribution will be to the health of consumers. On the other hand, it should not be ignored that SFAs also increase serum cholesterol levels.

The PUFA/SFA ratio from this study (1.62/1) and the essential fatty acids quantities in hare meat are favorable. Among polyunsaturated fatty acids, α-Linolenic acid and Eicosapentaenoic acid, Docosapentaenoic acid, and Docosahexaenoic acid receive great consideration based on their key role in human health [1,72,73,74,75,76,77,78,79,80], such as by lowering the amount of triglycerides in the blood and also aiding in cardiovascular illness prevention [1]. Consumption of EPAs and DHAs may ameliorate inflammatory processes in the body and decrease the frequency of allergic diseases in children. Also, EPAs and DHAs may have a positive role in fetal development and neuronal, retinal, and immune functions. EPAs and DHAs might also influence tumor cell proliferation [1]; in particular, DHA can promote tumor cell apoptosis, potentially inducing an oxidative process [1,77,78,79,80,81,82,83,84]. Excessive amounts of n-6 PUFAs and a high n-6/n-3 ratio stimulate the pathogenesis of many illnesses, like cancer and cardiac, vascular, autoimmune, and inflammatory diseases. High levels of n-3 PUFAs decrease these negative effects on human health. For the prevention of cardiovascular illness, a ratio of 4/1 can lead to a reduction in the death rate by 70%. Moreover, a ratio of 2.5/1 lowers the proliferation of rectal cancerous cells and the risk for developing breast cancer; an n-6/n-3 ratio of 2–3/1 decreases the symptoms for rheumatoid arthritis; and a ratio of 5/1 has a positive influence on asthma while a ratio of 10/1 has negative effects [80,84,85,86,87,88]. In hare meat, our original findings for the n-6/n-3 ratio (5.34/1) depict a great ratio for improving human health.

For humans, EFA is important because it cannot be innately produced and must be procured through the food consumed [1]. EFAs and DFAs have major beneficial effects, highlighted by their biological activity in humans, being involved in multiple metabolic functions. In other studies, the quantities of DFA in the breast fillets and thighs of different breeds of poultry [56] varied from 65.15% to 69.83% and 70.23% to 72.25%, respectively. On this subject, Banskalieva et al. [57] explain the potential beneficial health effects of DFA and the importance of multiple types of lipids for the health status of humans [38].

### 4.2. The Instrumental, Histological, and Sensory Evaluation of Hare Meat

The instrumental assessment of hare meat follows the texture and color parameters, compared by gender and muscle type.

#### 4.2.1. The Textural Indicators of Hare Meat

The highest mean value of the total shear force of hare meat (Table 5) was observed for SM muscles in females (4.32 kg/cm^2^), while the males had a mean shear force value of 3.29 kg/cm^2^ for the same muscle. The LD muscles of males required a stronger shear force (2.72 kg/cm^2^) compared with a force of 2.01 kg/cm^2^ for LD muscles of females (*p* < 0.001). The average values for the shear force are in line with other studies that analyze the textural traits of meat [15,17]. Compared to Razmaite and Šiukščius, 2023, the results did not show significant gender differences [17]. The shear force was higher only when they compared LD and BF muscles [17]. Other authors [15] obtained mean shear forces that varied from 2.97 to 4.02 kg/cm^2^ in sub-adult and adult hares and from 3.45 to 3.54 kg/cm^2^ by gender.

The age at slaughter, along with a multitude of other factors (the place of origin; dietary specificity; environmental factors; the temperature, humidity, ventilation of the environment; and instrumental methods used where the sample is prepared), influences the textural quality of hare meat. For other species, the shear force had similar mean values of 3.2–3.7 kg/cm^2^ in lamb [88] and close values for pork (5.2 kg/cm^2^) [87] and beef (5.4 kg/cm^2^) [86]. In our study, hare meat had a tenderness close to that of lamb, but with the specification that the meat quality is very variable, being influenced by multiple factors, as described above.

#### 4.2.2. The Color Indicators of Hare Meat

The color traits of LD samples (Table 6) had similar values for L*/lightness in males (28.54) and females (28.55). The values of brightness were lower than in other studies from Lithuania [17] (an average of 35.27 for LD muscles and of 37.61 for BF muscles).

In a study from Slovakia [13], the L* value, indicating the color of the meat, was significantly higher (*p* ≤ 0.05) in the meat of male hares (30.61) compared with meat from females (29.05), which is markedly darker. The same phenomenon was observed in the present study for male hares from the N-E of Romania. In other studies, the determined average values of L* for hare meat were higher (from 34.4 [15] to 37.61 [17]) compared with what we found (32.11 in SM and 28.94 in LD muscles).

The proportion of myoglobin and the type of myocytes contributed to the high redness (a* index) of meat, although it could be affected by exercise, alimentation, and climatic factors [56,83]. In the wild, male and female hares (*Lepus europaeus*) are not differentiated by their weight or body characteristics [81], and it would be surprising if the redness of the flesh would be a tool providing information about the gender of the animal by the specificity of the color of the meat.

**Figure 4:**
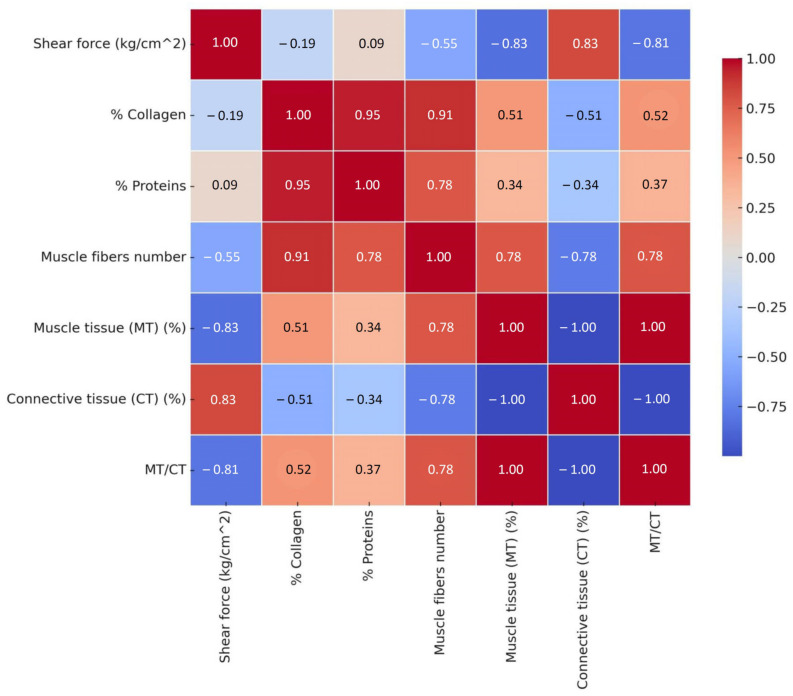
The statistical correlation of instrumental/textural, chemical, and histological analyses of hare meat by gender. Values close to 1 (dark red): strong positive correlation—when one value is higher, the other tends to increase. Values close to −1 (dark blue): strong negative correlation—when one value is higher, the other tends to decrease. Values close to 0 (pale shades): weak or no relationship.

The statistical correlation of instrumental/textural, chemical, and histological analysis of hare meat by gender (Figure 4) highlighted the following results:

1. Shear force (kg/cm²) is strongly negatively correlated with % MT (−0.83), where more muscle tissue reduces cutting force; connective tissue (CT) (%) (−0.83), where more connective tissue reduces cutting force; and MT/CT (−0.81), where a high ratio of muscle to connective tissue is associated with lower cutting force. It is not significantly correlated with % Collagen (−0.19) or % Proteins (0.09).
2. Collagen % has a very strong positive correlation with Proteins % (0.95), where a higher collagen content is associated with more proteins, and the number of muscle fibers (0.91), where a higher number of muscle fibers is associated with more collagen. It shares a weak negative relationship with shear force (−0.19).
3. Proteins % is strongly positively correlated with the number of muscle fibers (0.78), where more muscle fibers leads to a higher protein content. It has a weak or medium correlation with other variables such as MT/CT (0.37).
4. The number of muscle fibers is strongly positively correlated with % Collagen (0.91) and muscle tissue (MT) (%) (0.78), where more muscle fibers is associated with a greater proportion of muscle tissue. It is strongly negatively correlated with connective tissue (CT) (%) (−0.78), where more muscle fibers is associated with less connective tissue.
5. Muscle tissue (MT) (%) is strongly negatively correlated with connective tissue (CT) (%) (−1.00), sharing a perfectly inverse relationship. It is strongly positively correlated with MT/CT (1.00), as this is the ratio of muscle to connective tissues.
6. Connective tissue (CT) (%) is perfectly negatively correlated with muscle tissue (MT) (%) (−1.00), where more connective tissue indicates less muscle tissue. It is negatively correlated with MT/CT (−1.00), as a higher MT/CT ratio indicates less connective tissue.
7. MT/CT has identical correlations with the variables muscle tissue (MT) (%) and connective tissue (CT) (%), having perfectly opposite relationships with them.

For example, in this study, on average, the shear force was 3.08 kg/cm^2^, the collagen was 4.28%, and connective tissue was 32.71%, with inversely proportional correlations being observed between these parameters.

The shear force is mainly influenced by the ratio between contractile muscle cells and connective matrix of the muscle. The proportion of collagen and protein is deeply connected to the properties and number of muscle fibers. The number of contractile cells and the connective matrix are inversely proportional, and their ratio is a key indicator for the textural and mechanical properties of the analyzed samples.

### 4.3. The Sensory Indicators of Hare Meat

The average values of sensory indicators of LD and SM samples of hare meat are summarized in Figure 2 and Figure 3.

Hare meat was reported in other studies to have a darker color [8], to be more tender, and as having a moderate gamey flavor (4.65 to 6 points), showing little variation between samples. Hare meat’s sensory evaluation is poor overall [8]. An inconvenience in the comparison of sensory evaluations of hare meat is the small dimensions of the anatomical parts of this species [1] and the lack of data from the literature.

The obtained grades are difficult to interpret very precisely and objectively in terms of meat quality since we rely on differences of just one point (differences that can be somewhat subjective, depending on the opinion or sensory sensitivity of each taster) [1,8].

The results of the sensory evaluation were correlated with the instrumental and histological analysis. We noticed (in the sensory analysis) that when the fibrosity of samples was higher, the softness and succulence were lower for both LD and SM muscle groups.

**Figure 5:**
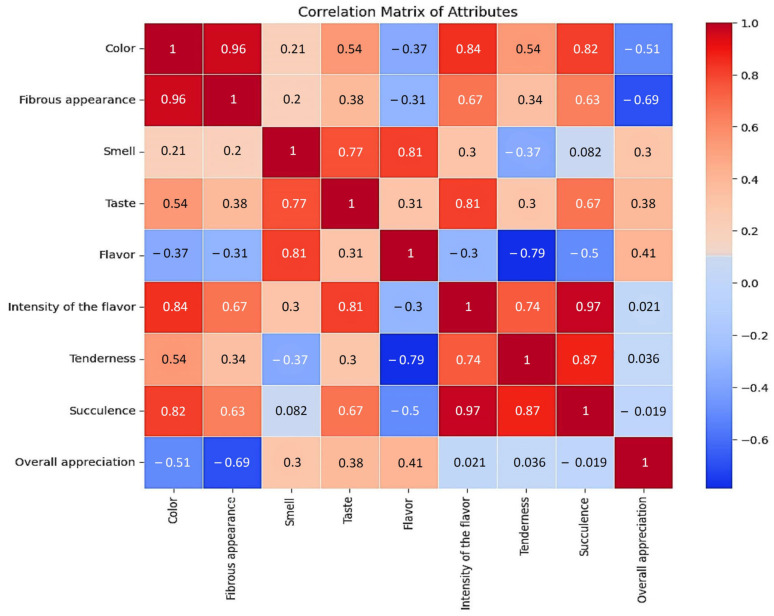
The statistical correlation of sensory analysis of hare meat by gender.

The correlation matrix applied for sensory characteristics shows the following relationships between different variables (Figure 5):

- The color and fibrous appearance have a very strong correlation (0.963), suggesting that as color increases, fibrous appearance also increases.
- The smell and taste have a moderate to strong correlation (0.773), indicating that smell and taste are related.
- The tenderness and succulence have a very strong correlation (0.872), suggesting that as tenderness increases, so does succulence.
- The flavor and tenderness have a strong negative correlation (−0.788), suggesting that as flavor increases, tenderness decreases.

The biological nutritional value of hare and rabbit meat is generally better than that of other game or domesticated species, based on the high content of valuable proteins (with essential amino acids), lipids with high amounts of PUFAs, and bioavailable vitamins and minerals [16,49].

Making a comparison for health lipid indices [1,4], in goose meat, the AI was 0.37 and the TI was 0.69 [82], which are smaller than the values in hare meat (AI = 0.72 and TI = 0.71). Hare meat presented lower values than those found in other meat species; for example, for rabbits from small breeds, the AI was 0.90 and the TI was 1.19 [83]. For poultry, the AI was 0.49 and the TI was 1.14 (in chicken) [84], and for turkey, the AI was 0.47 and the TI was 0.91 [85]. For other species, the health lipid indices were varied: for beef, the AI was 0.60 and the TI was 1.86 [86]; for pork, the AI was 0.47 and the TI was 1.12 [87]; and for lamb, the AI was 0.90 and the TI was 0.87 [1,4,88].

The practical implication of this study is that consumers should be able to interpret and comprehend the information about the quality of hare meat (e.g., the fatty acid composition, the healthy lipid values, and instrumental, histological, and sensory characteristics) without previously having tasted or purchased it.

## 5. Conclusions

The ΣPUFA/Σ SFA and the Σn6/n3 ratio was superior in male hares compared to female hares. The health lipid indices were better for males in general. Hedonic scoring was better in the LD muscles of males and SM muscles of females. A superior value of shear force was noticed in SM muscles for females compared to males. In LD muscles, the samples from males needed greater shear force than the samples from females (*p* < 0.001), suggesting a more appreciated sensory texture in the latter. For lightness (L*), greater average values were found for females compared to males. The red index (a*) was higher for females compared to males (*p* < 0.001), and the yellow index (b*) was higher in males than in females (*p* < 0.001). Meat from hares is valuable for human nutrition, meeting sensory, instrumental, and chemical proprieties and being preferable over the meat of farmed animals or other wild species (more valuable proteins, lower lipid content and energy content, and higher health lipid indices such as a better anti-hypercholesterol effect and higher polyunsaturation indices).

## Figures and Tables

**Table 1:** Five-point hedonic scale for sensory quality of hare meat (Ariño, 2007, adapted) [1,47].

Sensory Traits	Granted Scoring (Points)				
	1	2	3	4	5
Color	Extremely Pale	Pale	Pale red	Red	Intense red
Fibrous appearance	Weakly highlighted	Lightly highlighted	Medium highlighted	Distinctly highlighted	Strongly highlighted
Smell/rabbit odor	Imperceptible	Weakly perceptible	Medium perceptible	Distinct perceptible	Very Perceptible
Taste	Slightly unpleasant	No taste	Tasty enough	Tasty	Very tasty
Flavor	Slightly unpleasant	No flavor	Pleasant	Very pleasant	Extremely pleasant
Intensity of the flavor	Undetectable	Not enough flavor	Sufficiently pleasant	Pleasant and strong	Intense pleasant
Succulence	Dry	Insufficiently juicy	Sufficiently juicy	Juicy	Very juicy
Tenderness	Very stiff	Slightly stiff	Sufficiently soft	Soft	Very soft
Overall assessment	Unacceptable	Acceptable	Good	Very good	Exceptional

**Table 2:** The lipid, protein, and collagen content of hare meat (%).

Chemical Components	Muscles	Gender	Mean ± SEM		V%	*p* Value
Lipids	LD	M	1.52	0.13	10.69	0.1923 n.s.
		F	1.78	0.34	13.56	
	SM	M	1.81	0.13	11.12	0.9117 n.s.
		F	2.03	0.09	6.08	
Proteins	LD	M	21.67	0.05	0.71	0.0863 ns
		F	21.56	0.09	0.79	
	SM	M	21.64	0.06	0.43	0.0651 ns
		F	21.62	0.04	0.39	
Collagen	LD	M	4.26	0.12	1.51	0.1771 ns
		F	4.16	0.06	0.76	
	SM	M	4.32	0.08	0.64	0.3154 ns
		F	4.28	0.07	0.89	

LD—Longissimus dorsi; SM—Semimembranosus; SEM—standard error of mean; V%—coefficient of variation. Student test: ns = not significant.

**Table 3:** The fatty acids content (mg/100 g) in LD and SM muscles.

Fatty Acids		M/F *	LD				SM			
			Mean ± SEM		V%	*p*-Value	Mean ± SEM		V%	*p*-Value
SFA	C14:0	M	3.09	0.09	11.71	0.088 ns	1.13	0.04	15.4	0.239 ns
		F	1.22	0.04	14.15		1.21	0.04	14.22	
	C15:0	M	8.93	0.15	6.57	0.098 ns	7.97	0.19	9.47	0.003 **
		F	8.04	0.16	8.33		7.03	0.20	12.28	
	C16:0	M	322.87	10.75	12.9	0.588 ns	314.46	6.47	7.97	0.018 *
		F	338.98	5.74	7.18		280.13	8.03	12.16	
	C17:0	M	21.23	0.78	14.15	0.078 ns	18.89	0.74	15.13	3 × 10^−4^ ***
		F	16.91	0.33	8.29		14.06	0.39	11.63	
	C18:0	M	118.13	3.39	11.13	0.028 *	107.06	2.69	9.72	0.002 **
		F	100.04	1.57	6.67		92.11	2.40	11.04	
MUFA	C16:1n-7	M	1.05	0.02	8.45	0.092 ns	2.66	0.08	12.11	0.148 ns
		F	0.96	0.02	9.06		2.48	0.08	13.21	
	C18:1n-7	M	26.95	0.50	7.16	0.087 *	26.87	0.79	11.36	0.076 ns
		F	23.76	0.49	8.69		21.96	0.63	12.24	
	C18:1n-9	M	353.12	10.54	11.56	0.074 ns	308.08	7.29	9.16	0.003 **
		F	279.26	5.98	9.09		240.97	6.83	12.02	
PUFA	C18:2n-6	M	592.93	21.63	14.13	0.072 ns	557.02	24.08	16.74	3 × 10^−5^ ***
		F	502.04	9.51	8.04		414.16	8.52	8.73	
	C18:3n-3	M	53.16	2.51	18.31	0.252 ns	50.24	1.21	9.32	2 × 10^−5^ ***
		F	44.79	0.95	9.02		35.67	1.05	12.46	
	C20:2n-6	M	10.14	0.25	9.66	0.228 n.s	8.86	0.17	7.63	0.003 **
		F	9.06	0.18	8.41		8.14	0.27	14.16	
	C20:3n-6	M	1.87	0.09	19.62	5 × 10^−3^ ***	1.77	0.05	11.87	0.004 **
		F	1.98	0.10	21.18		1.13	0.03	12.75	
	C20:4n-6	M	63.28	3.23	19.78	0.005 **	61.27	2.24	14.18	0.217 ns
		F	67.35	2.21	13.94		63.54	1.79	11.95	
	C20:5n-3	M	4.12	0.16	15.22	0.008 **	3.12	0.15	18.35	0.875 ns
		F	2.89	0.12	17.58		3.18	0.14	18.09	
	C22:4n-6	M	17.76	0.92	20.03	0.375 ns	17.96	0.62	13.32	0.236 ns
		F	17.63	0.87	20.86		17.72	0.54	12.82	
	C22:5n-3	M	22.14	1.25	21.85	3 × 10^−6^ ***	21.22	0.89	16.24	0.778 ns
		F	25.02	1.29	21.82		21.46	0.85	16.81	
	C22:6n-3	M	42.63	2.15	19.53	0.214 ns	38.44	1.35	13.65	0.094 ns
		F	46.55	2.39	21.76		40.39	1.19	12.54	

LD—Longissimus dorsi; SM—Semimembranosus; SEM—standard error of mean; V%—coefficient of variation. Student test: ns = not significant, *p* > 0.05; * significant for *p* < 0.05; ** significant for *p* < 0.01; *** significant for *p* < 0.001.

**Table 4:** Total fatty acids (mg/100 g meat) and health lipid parameters for hare meat.

Health Lipid Parameters	Gender	Mean LD	*p* Value	Mean SM	*p* Value	Mean/Gender	Mean/Meat
Total SFAs	M	474.25	0.201 ns	449.51	0.01 **	461.88	445.87
	F	465.19		394.54		429.87	
Total MUFAs	M	381.12	<0.001 ***	337.61	0.005 **	359.37	322.03
	F	303.98		265.41		284.70	
Total PUFAs	M	808.03	* 0.05	759.90	0.001 ***	783.97	722.66
	F	717.31		605.39		661.35	
ΣPUFA n-6	M	685.98	0.30 ns	646.88	0.005 **	666.43	608.90
	F	598.06		504.69		551.38	
Σ PUFA n-3	M	122.05	0.65 ns	113.02	0.30 ns	117.54	113.76
	F	119.25		100.70		109.98	
ΣPUFAn6/n3	M	5.62	0.80 ns	5.72	0.25 ns	5.67	5.34
	F	5.02		5.01		5.01	
ΣPUFA/ΣSFA	M	1.70	0.85 ns	1.69	0.08 ns	1.70	1.62
	F	1.54		1.53		1.54	
PI	M	6.99	0.07 ns	6.58	0.01 **	6.78	6.09
	F	5.92		4.95		5.39	
DFAs	M	1307.28	0.05 *	1204.57	*** <001	1255.93	1149.02
	F	1121.33		962.91		1042.12	
%DFA	M	78.59	0.70 ns	77.86	0.33 ns	78.23	77.00
	F	75.44		76.10		75.77	
AI	M	0.81	0.01 **	0.76	<0.001 **	0.79	0.72
	F	0.72		0.61		0.66	
TI	M	0.77	0.17 ns	0.72	0.26 ns	0.75	0.71
	F	0.72		0.61		0.66	
h/H	M	3.64	0.54 ns	3.47	0.19 ns	3.56	3.30
	F	3.00		3.09		3.04	
NVI	M	1.54	0.37 ns	1.41	0.31 ns	1.47	1.35
	F	1.19		1.27		1.23	
Total fatty acids	M	1663.40	0.02 **	1547.02	0.001 ***	1605.21	1490.56
	F	1486.48		1265.34		1375.91	
EFAs	M	709.37	0.25 ns	668.53	0.005 ns	688.95	626.36
	F	614.18		513.37		563.775	
%EFA	M	42.65	0.85 ns	43.21	0.30 **	42.93	41.94
	F	41.32		40.57		40.94	

LD—Longissimus dorsi; SM—Semimembranosus. SFAs = saturated fatty acids; MUFAs = monounsaturated fatty acids; PUFAs = polyunsaturated fatty acids; EFAs = essential fatty acids; %EFA = EFA×100/ Σ Total fatty acids; DFA = desirable fatty acids; %DFA = DFA × 100/Σ Total fatty acids; PI = polyunsaturation index; AI = atherogenic index; TI = thrombogenic index; h/H = ratio between the hypocholesterolemic and hypercholesterolemic fatty acids; NVI = nutritive value index. Student test: ns = not significant, *p* > 0.05; * significant for *p* < 0.05; ** significant for *p* < 0.01; *** significant for *p* < 0.001.

**Table 5:** The textural traits of hare meat.

Muscles	Texture Indicators	Gender	Mean ± SEM		V%	*p* Value
LD	Shear force (kg/cm^2^)	F	2.01	0.06	13.59	0.000119 ***
		M	2.72	0.09	12.54	
	Firmness (kg/s × cm^2^)	F	0.92	0.02	11.46	0.02998231 *
		M	1.17	0.05	16.88	
	Area (kg × s/cm^2^)	F	3.95	0.13	14.48	0.125237 ns
		M	4.69	0.16	13.54	
SM	Shear force (kg/cm^2^)	F	4.32	0.15	14.53	9.07919 × 10^−5^ ***
		M	3.29	0.11	12.85	
	Firmness (kg/s × cm^2^)	F	1.54	0.04	11.87	0.036043 *
		M	1.16	0.05	16.43	
	Area (kg × s/cm^2^)	F	8.82	0.32	15.31	0.012539 *
		M	5.45	0.19	13.48	

LD—Longissimus dorsi; SM—Semimembranosus; SEM—standard error of mean; V%—coefficient of variation. Student test: ns = not significant, *p* > 0.05; * significant for *p* < 0.05; *** significant for *p* < 0.001.

**Table 6:** The color parameters of hare meat.

Muscles	Color Traits	Gender	Mean ± SEM		V%	*p* Value
LD	L*	F	28.55	0.86	12.72	0.232500 ns
		M	28.94	0.94	12.59	
	a*	F	13.58	0.40	12.65	0.000111 ***
		M	12.71	0.47	14.19	
	b*	F	12.77	0.51	16.86	0.109003 ns
		M	13.28	0.50	14.53	
SM	L*	F	32.11	1.24	16.38	0.089606 ns
		M	31.38	1.22	15.07	
	a*	F	6.84	0.30	18.83	0.382903 ns
		M	6.41	0.22	13.46	
	b*	F	10.02	0.28	12.03	0.506321 ns
		M	9.75	0.38	15.24	

LD—Longissimus dorsi; SM—Semimembranosus; SEM—standard error of mean; V%—coefficient of variation. Student test: ns = not significant, *p* > 0.05; *** significant for *p* < 0.001.

**Table 7:** The histological traits of hare meat.

Muscles	Histological Indicators	Gender	Mean	SEM	V%	*p* Value
LD	Large diameter (μ)	F	55.90	8.90	9.20	0.4235 ns
		M	57.33	12.89	11.15	
	Small diameter (μ)	F	20.28	3.50	10.10	0.8903 ns
		M	21.16	1.98	15.91	
	Mean diameter (μ)	F	46.69	4.31	8.30	0.8819 ns
		M	49.25	7.44	22.52	
	Ratio BD/sD (μ)	F	1.97	4.30	7.90	0.8038 ns
		M	2.71	6.52	0.74	
	Muscular fibers area (µ^2^)	F	1425.29	2.70	17.41	0.2357 ns
		M	1547.10	9.40	9.12	
	Cross-sectional area (µ^2^)	F	1,276,314.00	21.12	10.26	0.0613 ns
		M	2,321,564.00	27.12	14.33	
SM	Large diameter (μ)	F	69.7	13.5	7.5	0.0546 ns
		M	71.34	13.56	9.21	
	Small diameter (μ)	F	32.18	1.3	7.42	0.3758 ns
		M	34.28	2.2	14.62	
	Mean diameter (μ)	F	40.39	2.4	5.19	0.8038 ns
		M	40.81	7.88	11.91	
	Ratio BD/sD (μ)	F	3.92	2.3	1.31	0.0131 ns
		M	4.3	6.15	0.27	
	Muscular fibers area (µ^2^)	F	945.56	3.51	2.38	0.7726 ns
		M	989.42	2.74	3.12	
	Cross-sectional area (µ^2^)	F	756,432	1.14	3.75	0.5298 ns
		M	805,306	16.01	4.16	

BD = Large diameter; sD = Small diameter. LD—Longissimus dorsi; SM—Semimembranosus, SEM—standard error of mean; V%—coefficient of variation. Student test: ns = not significant, *p* > 0.05.

**Table 8:** The number of fibers and the types of muscle tissues in hares.

Muscles/ Gender	Muscle Fiber Number	*p*	Density	*p*	Muscle Tissue (MT) (%)	*p*	Connective Tissue (CT) (%)	*p*	MT/CT	*p*
LD/F	105.00	0.4235 ns	256.00	0.001 ***	69.40	0.6543 ns	30.61	0.6543 ns	2.27	0.8765ns
LD/M	115.00		257.00		71.01		28.99		2.45	
SM/F	99.00	0.5123 ns	289.00	0.8765 ns	63.82	0.7542 ns	36.18	0.7542 ns	1.76	0.9123ns
SM/M	107.00		263.00		64.92		35.08		1.85	

LD—Longissimus dorsi; SM—Semimembranosus. Density = muscle fibers/mm^2^ muscles. Student test: ns = not significant, *** significant for *p* < 0.001.

## Data Availability

The original contributions presented in this study are included in the article. Further inquiries can be directed to the corresponding authors.

## References

[1] Frunză G., Ciobanu M.M., Murariu O.C., Rațu R.N., Radu-Rusu R.-M., Simeanu C., Boișteanu P.-C. (2023). Effect of Gender and Muscle Type on Fatty Acid Profile, Sanogenic Indices, and Instrumental and Sensory Analysis of Flemish Giant Rabbit Meat. Agriculture.

[2] Boișteanu P.B., Flocea E.I., Anchidin B.G., Mădescu B.M., Matei M., Murariu O.C., Frunză G., Postolache N., Ciobanu M.M. (2024). Essential and Toxic Elements Analysis of Wild Boar Tissues from North-Eastern Romania and Health Risk Implications. Front. Sustain. Food Syst..

[3] Ciobanu M.M., Postolache A.N., Lipșa F.D., Munteanu M., Rațu R.N., Murariu O.C., Boișteanu P.C. (2022). Meat Fatty Acid Composition of Wild Boars Hunted in Romania in Relationship to Gender and Age-Class. Animals.

[4] Frunză G., Murariu O.C., Ciobanu M.-M., Radu-Rusu R.-M., Simeanu D., Boișteanu P.-C. (2023). Meat Quality in Rabbit (*Oryctolagus cuniculus*) and Hare (*Lepus europaeus* Pallas)—A Nutritional and Technological Perspective. Agriculture.

[5] Buglione M., de Filippo G., Conti P., Fulgione D. (2022). Eating in an extreme environment: Diet of the European hare (*Lepus europaeus*) on Vesuvius. Eur. Zool. J..

[6] Vizzarri F., Nardoia M., Palazzo M. (2014). Effect of dietary Lippia citriodora extract on productive performance and meat quality parameters in hares (*Lepus europaeus* Pall.). Arch. Anim. Breed..

[7] Rigo N., Trocino A., Poppi L., Giacomelli M., Grilli G., Piccirillo A. (2015). Performance and mortality of farmed hares. Animal.

[8] Rødbotten M., Kubberød E., Lea P., Ueland Ø. (2004). A sensory map of the meat universe. Sensory profile of meat from 15 species. Meat Sci..

[9] Konjevic D. (2007). Hare brown (*Lepus europaeus* Pallas) and potential in diet of people today. Prof. Work.

[10] Valencak T.G., Arnold W., Tataruch F., Ruf T. (2003). High content of polyunsaturated fatty acids in muscle phospholipids of a fast runner, the European brown hare (*Lepus europaeus*). J. Comp. Physiol..

[11] Valencak T.G., Gamsjäger L., Ohrnberger S., Culbert N.J., Ruf T. (2015). Healthy n-6/n-3 fatty acid composition from five European game meat species remains after cooking. BMC Res. Notes.

[12] Skrivanko M., Hadžiosmanovic M., Cvrtila Z., Zdolec N., Filipovic I., Kozacinski L., Florijanci´c T., Boškovic I. (2008). The hygiene and quality of hare meat (*Lepus europaeus* Pallas) from Eastern Croatia. Arch. Lebensm..

[13] Mertin D., Slamecka J., Ondruška I., Zaujec K., Jurcík R., Gašparík J. (2012). Comparison of meat quality between European brown hare and domestic rabbit. Slovak J. Anim. Sci..

[14] Strmiskova G., Strmiska F. (1992). Contents of mineral substances in venison. Food/Nahrung.

[15] Trocino A., Birolo M., Dabbou S., Gratta F., Rigo N., Xiccato G. (2018). Effect of age and gender on carcass traits and meat quality of farmed brown hares. Animal.

[16] Króliczewska B., Mista D., Korzeniowska M., Pecka-Kiełb E., Zachwieja A. (2018). Comparative evaluation of the quality and fatty acid profile of meat from brown hares and domestic rabbits offered the same diet. Meat Sci..

[17] Razmaitė V., Šiukščius A. (2023). Effects of Sex and Hunting Season on Carcass and Meat Quality Characteristics of the Brown Hare (*Lepus europaeus*). Foods.

[18] Schai-Braun S.C., Kowalczyk C., Klansek E., Hackländer K. (2019). Estimating sustainable harvest rates for European hare (*Lepus Europaeus*) populations. Sustainability.

[19] Jennings N., Smith R.K., Hackländer K., Harris S., White P.C.L. (2006). Variation in demography, condition and dietaryquality of hares Lepus europaeus from high-density and low-density populations. Wildl. Biol..

[20] Reichlin T., Klansek E., Hackländer K. (2006). Diet selection by hares (*Lepus europaeus*) in arable land and its implications for habitat management. Eur. J. Wildl. Res..

[21] Petrovan S.O., Ward A.I., Wheeler P.M. (2013). Habitat selection guiding agri-environmental schemes for a farmland specialist, the brown hare. Anim. Conserv..

[22] Gryz J., Krauze-Gryz D. (2022). Why Did Brown Hare Lepus europaeus Disappear from Some Areas in Central Poland?. Diversity.

[23] Viviano A., Mori E., Fattorini N., Mazza G., Lazzeri L., Panichi A., Strianese L., Mohamed W.F. (2021). Spatiotemporal overlap between the European brown hare and its potential predators and competitors. Animals.

[24] Petelis K., Brazaitis G. (2009). The European hare (*Lepus europaeus* Pallas) population in Lithuania: The status and causes of abundance change. Acta Biol..

[25] Vicenti A., Ragni M., di Summa A., Marsico G., Vonghia G. (2003). Influence of feeds and rearing system on the productive performances and the chemical and fatty acid composition of hare meat. Food Sci. Technol. Int..

[26] Bock A. (2020). Lepus europaeus (*Lagomorpha: Leporidae*). Mamm. Species.

[27] Ruf T., Valencak T., Tataruch F., Arnold W. (2006). Running Speed in Mammals Increases with Muscle n-6 Polyunsaturated Fatty Acid Content. PLoS ONE.

[28] García-Abad C.S., De La Varga M.E.A., Valle C.D., Lacasa V.R.G. (2012). An approach to the statistics of wild lagomorph captive rearing for releasing purposes in Spain. World Rabbit Sci..

[29] Kelava Ugarković N., Bedeković D., Greiner K., Fabijanić N., Prpić Z., Konjačić M. (2024). Carcass Characteristics and Meat Quality of Wild-Living Mallard (*Anas platyrhynchos* L.) Originating from Croatia. Foods.

[30] Papadomichelakis G., Zoidis E., Pappas A.C., Hadjigeorgiou I. (2017). Seasonal variations in the fatty acid composition of Greek wild rabbit meat. Meat Sci..

[31] FAO Stat. https://www.fao.org/faostat/en/#home.

[32] The Minister of Environment, Water and Forests. https://www.mmediu.ro/articol/ordinul-ministrului-mediului-apelor-si-padurilor-nr-1571-07-06-2022-privind-aprobarea-cotelor-de-recolta-pentru-unele-specii-de-fauna-de-interes-cinegetic-la-care-vanatoarea-este-permisa-pentru-perioada-iunie-2022-14-mai-2023-precum-si-a-anexelor-1-6-ale-a/5244.

[33] Stroh G. (1931). Zwei sichere Altersmerkmale beim Hasen. Berliner. Tierärztl. Wschr..

[34] Prevolnik M., Čandek Potokar M., Škorjanc D. (2004). Ability of NIR spectroscopy to predict meat chemical composition and quality—A review. Czech J. Anim. Sci..

[35] Pla M., Hernández P., Ariño B., Ramírez J.A., Díaz I. (2007). Prediction of fatty acid content in rabbit meat and discrimination between conventional and organic production systems by NIRS methodology. Food Chem..

[36] Chen Y., Qiao Y., Xiao Y., Chen H., Zhao L., Huang M., Zhou G. (2016). Differences in physicochemical and nutritional properties of breast and thigh meat from crossbred chickens, commercial broilers and spent hens. Asian-Australas. J. Anim. Sci..

[37] Timmons J.S., Weiss W.P., Palmquist D.L., Harper W.J. (2001). Relationships among dietary roasted soybeans, milk components, and spontaneous oxidized flavor of milk. J. Dairy Sci..

[38] Ulbricht T.L.V., Southgate D.A.T. (1991). Coronary heart disease: Seven dietary factors. Lancet.

[39] Fernandez M., Ordóñez J.A., Cambero I., Santos C., Pin C., De La Hoz L. (2007). Fatty acid compositions of selected varieties of Spanish dry ham related to their nutritional implications. Food. Chem..

[40] Mierliță D., Pop I.M., Lup F., Simeanu D., Vicas S.I., Simeanu C. (2018). The Fatty Acids Composition and Health Lipid Indices in the Sheep Raw Milk Under a Pasture-Based Dairy System. Rev. Chim..

[41] Struți D.I., Mierliță D., Simeanu D., Pop I.M., Socol C.T., Papuc T., Macri A.M. (2020). The effect of dehulling lupine seeds (*Lupinus albus* l.) from low-alkaloid varieties on the chemical composition and fatty acids content. Rev. Chim..

[42] Abdel-Naeem H.H., Sallam K.I., Zaki H.M. (2021). Effect of different cooking methods of rabbit meat on topographical changes, physicochemical characteristics, fatty acids profile, microbial quality, and sensory attributes. Meat Sci..

[43] Dal Bosco A., Cartoni Mancinelli A., Vaudo G., Cavallo M., Castellini C., Mattioli S. (2022). Indexing of Fatty Acids in Poultry Meat for Its Characterization in Healthy Human Nutrition: A Comprehensive Application of the Scientific Literature and New Proposals. Nutrients.

[44] Murariu O.C., Murariu F., Frunză G., Ciobanu M.M., Boișteanu P.C. (2023). Fatty Acid Indices and the Nutritional Properties of Karakul Sheep Meat. Nutrients.

[45] Wereńska M., Haraf G., Wołoszyn J., Goluch Z., Okruszek A., Teleszko M. (2021). Fatty acid profile and health lipid indicies of goose meat in relation to various types of heat treatment. Poult. Sci..

[46] Ariño B., Hernández P., Pla M., Blasco A. (2007). Comparison between rabbit lines for sensory meat quality. Meat Sci..

[47] Dal Bosco A., Cavallo M., Menchetti L., Angelucci E., Cartoni Mancinelli A., Vaudo G., Marconi S., Camilli E., Galli F., Castellini C. (2024). The Healthy Fatty Index Allows for Deeper Insights into the Lipid Composition of Foods of Animal Origin When Compared with the Atherogenic and Thrombogenicity Indexes. Foods.

[48] Carpenè E., Andreani G., Ferlizza E., Menotta S., Fedrizzi G., Isani G. (2020). Trace Elements in Home-Processed Food Obtained from Unconventional Animals. Life.

[49] Dalle Zotte A., Szendro Z. (2011). The role of rabbit meat as functional food. Meat Sci..

[50] Alves dos Santos J.J., Fonseca Pascoal L.A., Brandão Grisi C.V., Costa Santos V., Santana Neto D.C., Filho J.J., Ferreira Herminio M.P., Fabricio Dantas A. (2022). Soybean oil and selenium yeast levels in the diet of rabbits on performance, fatty acid profile, enzyme activity and oxidative stability of meat. Livest. Sci..

[51] Bouzaida M.D., Resconi V.C., Gimeno D., Romero J.V., Calanche J.B., Barahona M., Olleta J.L., María G.A. (2021). Effect of Dietary Grape Pomace on Fattening Rabbit Performance, Fatty Acid Composition, and Shelf Life of Meat. Antioxidants.

[52] Guedes C.M., Almeida M., Closson M., Garcia-Santos S., Lorenzo J.M., Domínguez R., Ferreira L., Trindade H., Silva S., Pinheiro V. (2022). Effect of Total Replacement of Soya Bean Meal by Whole Lupine Seeds and of Gender on the Meat Quality and Fatty Acids Profile of Growing Rabbits. Foods.

[53] Hernandez P., Cesari V., Blasco A. (2008). Effect of genetic rabbit lines on lipid content, lipolytic activities and fatty acid composition of hind leg meat and perirenal fat. Meat Sci..

[54] Needham T., Bureš D., Černý J., Hoffman L.C. (2023). Overview of game meat utilisation challenges and opportunities: A European perspective. Meat Sci..

[55] European Federation of Associations for Hunting (FACE) (2021). FACE Annual Report 2021. https://www.face.eu/about-face/.

[56] Rikimaru K., Takahashi H. (2010). Evaluation of the meat from Hinaijidori chickens and broilers: Analysis of general biochemical components, free amino acids, inosine 5′-monophosphate, and fatty acids. J. Appl. Poult. Res..

[57] Banskalieva V., Sahlu T., Goetsch A. (2000). Fatty acid composition of goat muscles and fat depots: A review. Small Rumin. Res..

[58] Fernandes C.E., Vasconcelos M.A.D.S., De Almeida Ribeiro M., Sarubbo L.A., Andrade S.A.C., Filho A.B.D.M. (2014). Nutritional and Lipid Profiles in Marine Fish Species from Brazil. Food Chem..

[59] Kumar S.A., Kim H.J., Jayasena D.D., Jo C. (2023). On-Farm and Processing Factors Affecting Rabbit Carcass and Meat Quality Attributes. Food Sci. Anim. Res..

[60] Dal Bosco A., Mugnai C., Roscini V., Mattioli S., Ruggeri S., Castellini C. (2014). Effect of Dietary Alfalfa on the Fatty Acid Composition and Indexes of Lipid Metabolism of Rabbit Meat. Meat Sci..

[61] Dabbou S., Gai F., Renna M., Rotolo L., Dabbou S., Lussiana C., Kovitvadhi A., Brugiapaglia A., De Marco M., Helal A.N. (2017). Inclusion of bilberry pomace in rabbit diets: Effects on carcass characteristics and meat quality. Meat Sci..

[62] D’Agata M., Preziuso G., Russo C., Dalle Zotte A., Mourvaki E., Paci G. (2009). Effect of an outdoor rearing system on the welfare, growth performance, carcass and meat quality of a slow-growing rabbit population. Meat Sci..

[63] Rasinska E., Czarniecka-Skubina E., Rutkowska J. (2018). Fatty acid and lipid contents differentiation in cuts of rabbit meat. CyTA J. Food.

[64] Hoffman L.C., van Schalkwyk D.L., Muller M., Needham T., McMillin K.W. (2021). Carcass Yields and Physical-Chemical Meat Quality Characteristics of Namibian Red Hartebeest (*Alcelaphus buselaphus*) as Influenced by Sex and Muscle. Foods.

[65] Ciobanu M.M., Munteanu M., Postolache A.N., Boișteanu P.C. (2020). Toxic heavy metals content in wild boar and venison meat: A brief review. Sci. Pap. Ser. D Anim. Sci..

[66] Hernández P., Zotte A.D., de Blas C., Wiseman J. (2010). Influence of diet on rabbit meat quality. Nutrition of the Rabbit.

[67] Dalle Zotte A. (2002). Perception of Rabbit Meat Quality and Major Factors Influencing the Rabbit Carcass and Meat Quality. Livest. Prod. Sci..

[68] Willett W.C. (2012). Dietary fats and coronary heart disease. J. Intern. Med..

[69] Brukało K.M., Nowak J., Pietrzykowska A., Fras N., Ožbolt P., Kowalski O., Blenkuš M.G. (2024). Public food procurement as a tool of sustainable food and nutrition policy—Fat products. Front. Sustain. Food Syst..

[70] Lescinsky H., Afshin A., Ashbaugh C., Bisignano C., Brauer M., Ferrara G., Hay S.I., He J., Iannucci V., Marczak L.B. (2022). Health effects associated with consumption of unprocessed red meat: A Burden of Proof study. Nat. Med..

[71] Chen J., Liu H. (2020). Nutritional indices for assessing fatty acids: A mini-review. Int. J. Mol. Sci..

[72] Grosso G. (2023). Role of food processing on human health and current limitations. Int. J. Food Sci. Nutr..

[73] Liu H.W., Gai F., Gasco L., Brugiapaglia A., Lussiana C., Guo K.J., Tong J.M., Zaccarato I. (2009). Effects of chestnut tannins on carcass characteristics, meat quality, lipid oxidation and fatty acid composition of rabbits. Meat Sci..

[74] Capra G., Martínez R., Fradiletti F., Cozzano S., Repiso L., Márquez R., Ibáñez F. (2013). Meat quality of rabbits reared with two different feeding strategies: With or without fresh alfalfa ad libitum. World Rabbit. Sci..

[75] Dal Bosco A., Gerencsér Z., Szendrő Z., Mugnai C., Cullere M., Kovàcs M., Ruggeri S., Mattioli S., Castellini C., Dalle Zotte A. (2014). Effect of dietary supplementation of Spirulina (*Arthrospira platensis*) and Thyme (*Thymus vulgaris*) on rabbit meat appearance, oxidative stability and fatty acid profile during retail display. Meat Sci..

[76] Dal Bosco A., Castellini C., Martino M., Mattioli S., Marconi O., Sileoni V., Ruggeri S., Tei F., Benincasa P. (2015). The effect of dietary alfalfa and flax sprouts on rabbit meat antioxidant content, lipid oxidation and fatty acid composition. Meat Sci..

[77] Simopoulos A.P. (2016). An Increase in the Omega-6/Omega-3 Fatty Acid Ratio Increases the Risk for Obesity. Nutrients.

[78] Alonso-Vale M.I., Cruz M., Bolsoni-Lopes A., Sa Paula de Andrade R. (2015). Palmitoleic Acid (C16:1n7) Treatment Enhances Fatty Acid Oxidation and Oxygen Consumption in White Adipocytes. Biochem. Mol. Biol..

[79] Betz I.R., Qaiyumi S.J., Goeritzer M., Thiele A., Brix S., Beyhoff N., Grune J., Klopfleisch R., Greulich F., Uhlenhaut N.H. (2021). Cardioprotective Effects of Palmitoleic Acid (C16:1n7) in a Mouse Model of Catecholamine-Induced Cardiac Damage Are Mediated by PPAR Activation. Int. J. Mol. Sci..

[80] Simopoulos A.P. (2002). The importance of the ratio of omega-6/omega-3 essential fatty acids. Biomed. Pharmacother..

[81] Trocchi V., Riga F. (2005). I Lagomorfi in Italia. Linee guida per la conservazione e la gestione. Min. Politiche Agric. For.–Ist. Naz. Fauna Selvatica Doc. Tec..

[82] Wołoszyn J., Haraf G., Okruszek A., Werenska M., Goluch Z., Teleszko M. (2020). Fatty acid profiles and health lipid indices in the breast muscles of local Polish goose varieties. Poult. Sci..

[83] Dal Bosco A.D., Castellini C., Bernardini M. (2001). Nutritional quality of rabbit meat as affected by cooking procedure and dietary vitamin. Eur. J. Food Sci..

[84] Puerto M., Cabrera M.C., Saadoun A. (2017). A note of fatty acids profile of meat from broiler chickens supplemented with inorganic or organic selenium. Int. J. Food Sci..

[85] Skiepko N., Chwastowska-Siwecka I., Kondratowicz J., Mikulski D. (2016). Fatty acid profile, total cholesterol, vitamin content TBARS value of Turkey breast muscle cured with the addition lycopene. Poult. Sci..

[86] Mapiye C., Chimonyo M., Dzama K., Hugo A., Strydom P.E., Muchenje V. (2011). Fatty acid composition of beef from Nguni Steers supplemented with Acacia karroo leaf-meal. J. Food Compos. Anal..

[87] Kasprzyk A., Tyra M., Babicz M. (2015). Fatty acid profile of pork from a local and a commercial breed. Arch. Anim. Breed..

[88] Margetín M., Apolen D., Oravcová M., Vavrišínová K.L.A., Peškovičová D., Luptáková L., Krupová Z., Bučko O., Blaško J. (2014). Fatty acids profile of intramuscular fat in light lambs traditionally and artificially reared. J. Cent. Eur. Agric..

